# Prediagnostic serum 25-hydroxyvitamin D and melanoma risk

**DOI:** 10.1038/s41598-020-77155-2

**Published:** 2020-11-18

**Authors:** Jo S. Stenehjem, Nathalie C. Støer, Reza Ghiasvand, Tom K. Grimsrud, Ronnie Babigumira, Judy R. Rees, Lill Tove Nilsen, Bjørn Johnsen, Per M. Thorsby, Marit B. Veierød, Trude E. Robsahm

**Affiliations:** 1 Department of Biostatistics, Oslo Centre for Biostatistics and Epidemiology, University of Oslo, Blindern, P.O. Box 1122, 0317 Oslo, Norway; 2 Department of Research, Cancer Registry of Norway, Oslo, Norway; 3 Department of Research and Development, Division of Emergencies and Critical Care, Oslo University Hospital, Oslo, Norway; 4 Oslo Centre for Biostatistics and Epidemiology, Oslo University Hospital, Oslo, Norway; 5 New Hampshire State Cancer Registry, Lebanon, NH USA; 6 Department of Epidemiology, Geisel School of Medicine at Dartmouth, Lebanon, NH USA; 7 Norwegian Radiation and Nuclear Safety Authority, Østerås, Norway; 8 Hormone Laboratory, Department of Medical Biochemistry, Oslo University Hospital, Oslo, Norway

**Keywords:** Cancer epidemiology, Biomarkers

## Abstract

Previous studies of serum 25-hydroxyvitamin D (25(OH)D) in relation to melanoma have shown conflicting results. We conducted a nested case–control study of 708 cases and 708 controls, using prediagnostically collected serum, to study 25(OH)D and melanoma risk in the population-based Janus Serum Bank Cohort. Stratified Cox regression was used to estimate hazard ratios (HRs) with 95% confidence intervals (CIs) adjusted for ultraviolet radiation (UVR) indicators and stratified by ambient UVB of residence and body mass index (BMI). Non-linear associations were studied by restricted cubic splines. Missing data were handled with multiple imputation by chained equations. We found an HR of melanoma risk of 1.01 (95% CI: 0.99, 1.04) and an HR_imputed_ of 1.02 (95% CI: 1.00, 1.04) per 5-nmol/L increase. The spline model showed exposure-risk curves with significantly reduced melanoma risk between 60 and 85 nmol/L 25(OH)D (reference 50 nmol/L). Non-significant J-shaped curves were found in sub-analyses of subjects with high ambient UVB of residence and of subjects with BMI < 25 kg/m^2^. Our data did not yield persuasive evidence for an association between 25(OH)D and melanoma risk overall. Serum levels within the medium range might be associated with reduced risk, an association possibly mediated by BMI.

## Introduction

Incidence and mortality rates of cutaneous melanoma (hereafter melanoma) are increasing in fair-skinned populations worldwide^1^. Melanoma is currently the third and fifth most frequent cancer in Europe and USA, respectively^1,2^. Ultraviolet radiation (UVR) exposure is the primary environmental cause of melanoma^3^, and has been estimated to account for 80–90% of all cases^4–6^.

UVR exposure is also the main source of vitamin D^7^. Since the 1980s, vitamin D has been hypothesized to reduce both cancer incidence and mortality^8^. Laboratory studies have demonstrated anticancer properties of the hormonal form of vitamin D, 1,25-dihydroxycholecalciferol (1,25(OH)_2_D) in melanoma cell lines^9–11^. At UVR wavelengths of 290–320 nm, 7-dehydrocholesterol in the keratinocytes is converted to previtamin D3 (cholecalciferol). Previtamin D3 and D2 (ergocalciferol) are then hydroxylated in the liver to 25-hydroxyvitamin D (25(OH)D), representing the circulating form of vitamin D. The hormonal form, 1,25(OH)_2_D, is primarily formed in the kidney through endocrine synthesis (classical synthesis), but may also be formed in the skin and in non-renal cells by conversion of 25(OH)D to 1,25(OH)_2_D in an autocrine manner (non-classical synthesis), which has given support also for a role of 25(OH)D in cancer prevention^12–16^. Low 25(OH)D levels have been associated with obesity due to the fat-soluble properties of 25(OH)D,^17–19^ suggesting that body mass index (BMI) should be included as a factor when examining the association between 25(OH)D and melanoma association.

Although laboratory studies provide a biological plausibility that an adequate level of 25(OH)D may inhibit cancer development, some recent studies have not found evidence of an association^20,21^, while others have reported an inverse association between 25(OH)D and cancer risk^22,23^. The three most recent meta-analyses of serum 25(OH)D and melanoma risk concluded that there is no association^24–26^. However, the two earlier meta-analyses did not discriminate between pre- and postdiagnostic sampling of sera^24,26^, while the most recent only included prospective studies^25^. Studies with postdiagnostic serum 25(OH)D have generally shown lower 25(OH)D concentrations in melanoma patients than in controls^27–30^, but these findings might be due to reverse causation by the carcinogenic process itself^31–33^. Prospective studies have reported increased melanoma risk with increasing prediagnostic 25(OH)D serum levels^34–38^, most likely due to confounding by UVR exposure^25,35,38^. However, the prospective studies also vary in sample size, adjustment for UVR exposure, whether both sexes were studied, previous cancer history, and whether the melanoma cases were invasive or in situ*.* To our knowledge, no study has prospectively examined the 25(OH)D-melanoma association by ambient UVB of residence, BMI, anatomical site and histological subtype.

In the present study, we used stored sera from the population-based and prospective Janus Serum Bank Cohort (hereafter Janus Cohort) to examine the association between prediagnostic 25(OH)D and melanoma risk with adjustment for UVR indicators. Further, we aimed to assess the 25(OH)D-melanoma association with non-linear models, and by stratification on ambient UVB of residence, BMI, anatomical site and histological subtype.

## Methods

### Study population

We performed a case–control study nested in the Janus Cohort, a population-based biobank for prospective cancer studies containing serum samples from 318,628 Norwegians collected 1972–2003. Anthropometric measurements and questionnaire data are available for 292,851 cohort members who also participated in at least one of five regional health surveys. Detailed descriptions of the cohort establishment and data have been published elsewhere^39,40^.

The present study was designed as a nested case–control study within the prospective Janus Cohort to reduce laboratory costs. No samples were analyzed for 25(OH)D immediately after blood draw, but stored at − 25 °C and thawed in 2016–2017 for the cases and controls selected for this study according to a pre-defined study protocol^33^. The design and analysis-time of this study is therefore prospective since all study participants were cancer free at blood-draw. The study research file was created by linkage of the Janus Cohort to Statistics Norway, the Norwegian National Population Register and the Cancer Registry of Norway (CRN) by the use of the 11-digit personal identification number assigned to all Norwegian citizens. Additionally it contains group level information on ambient ultraviolet-B (UVB) of residence and sun tanning behavior from the Norwegian Women and Cancer (NOWAC) cohort study. A study protocol with details about the linkage and data sources for the current study has been published^33^.

Legal and ethical approval was obtained from the Regional Committee for Medical Research Ethics.

### Identification of cancer cases

The linkage to the Norwegian National Registry (Population Register) provided information on vital status, year of death or year of emigration. The CRN linkage provided a complete cancer history (1953–2009) for all individuals with a melanoma diagnosis. Reporting of incident cancers to the CRN is compulsory by law, and data from several sources ensure high quality data with 99.5% being morphologically verified^41,42^. Cases were required to (1) be histologically verified melanomas, (2) have no cancer before their melanoma diagnosis (except basal cell carcinoma, which is not registered in the CRN), (3) be diagnosed after recruitment into the Janus Cohort, (4) be aged < 75 years at diagnosis, (5) be diagnosed before 2009 and (6) have at least 2 years between time from blood draw and subsequent diagnosis^33^. Information on tumour localization was based on a local CRN modified version of the International Classification of Diseases, 7th revision (ICD-7 codes 1900–1909), converted into ICD-10 codes (head and neck = C43.0–4; trunk = C43.5; upper limbs = C43.6; lower limbs = C43.7; other and not otherwise specified = C43.8–9). Histological subtypes of melanoma were defined by codes from ICD-Oncology, 3rd edition (superficial spreading melanoma = 8743; nodular melanoma = 8721; other = 8000, 8723, 8730, 8742, 8744, 8745, 8770, 8772; not otherwise specified = 8720).

**Figure 1:**
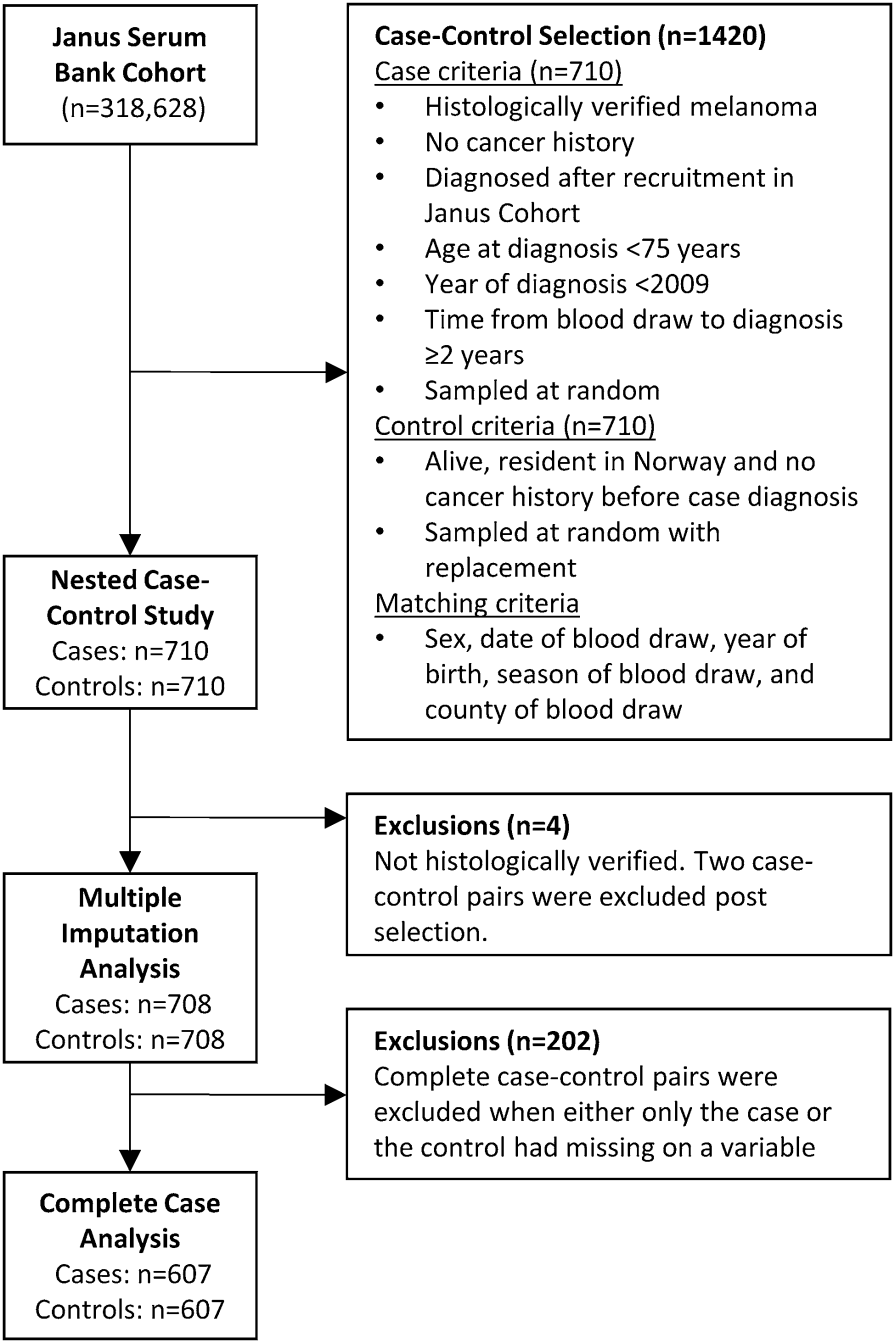
Overview of study design (selection of cases and controls) and exclusions.

Follow-up was defined as from the year recruited into the Janus Cohort (baseline, ranging 1972–2003) through 31 December 2009. During follow-up, a total of 1810 incident melanoma cases were identified (using the abovementioned case criteria). The number of included cases was limited to a random selection of 710 of the identified melanomas, based on power calculations to reduce laboratory costs^33^. A flow chart of the study design and exclusions is shown in Fig. 1. Two cases were excluded (together with their controls) post-selection as they were not histologically verified (Fig. 1).

### Identification of controls

Controls were drawn at random with replacement from a pool of available controls according to an incidence density sampling scheme^43^, and required to be alive, resident in Norway and without a cancer history before the melanoma diagnosis of the case. One control was matched to each case on sex, date of blood draw (+ /− 2 years), year of birth (+ /− 1 year), season of blood draw to account for seasonal variation in 25(OH)D (December–February, March–May, June–August, September–November)^44^, and county of blood draw.

### Assessment of exposures

#### Laboratory analyses

Concentrations of 25(OH)D were measured during 2016–2017 using an in-house developed liquid chromatography/tandem mass spectrometry method at the Hormone Laboratory, Oslo University Hospital^45^. The laboratory participates in the Vitamin D External Quality Assessment Scheme for total 25(OH)D. Further, the laboratory is approved by Norwegian Accreditation and complies with the requirements of the NS-EN ISO/IEC 17025 standards. Laboratory results of 25(OH)D were provided in nmol/L on a continuous scale. We categorized 25(OH)D into quantiles based on the 25(OH)D distribution among controls (quintile 1: < 49.2, quintile 2: 49.2–61.8, quintile 3: 61.9–74.7, quintile 4: 74.8–89.2, quintile 5: > 89.2; and tertiles in sub-analyses tertile 1: < 58.5 tertile 2: 58.5–79.8, tertile 3: > 79.8 nmol/L), and according to clinical cut points (11–29, 30–49, 50–74, 75–212 nmol/L).

#### Health survey and registry data

Baseline measurements of height (to the nearest 1 cm) and weight (to the nearest 0.5 kg) were obtained by trained staff according to a standardized protocol. BMI was calculated (weight/height^2^). Body surface area (BSA, m^2^) was calculated using the DuBois and DuBois’ equation (weight^0.4253^ × height^0.7253^ × 0.007184)^46^.

 The variable ‘Ambient UVB of residence’ at baseline was constructed by categorizing county of residence into north, mid, southwest, southeast inland, and southeast coast (in an ascending order from lower to higher UVB doses). The variable ‘Lifetime ambient UVB’ was constructed by linking county-specific UVB doses to county of residence at baseline, and then by cumulating yearly UVB doses from birth to diagnosis for each case–control set. UVB data were derived from UV measurement stations and from modelled values as described by Medhaug et al.^47^.

The Janus Cohort lacked individual data on UVR behavior variables. Therefore, group level data on annual mean number of sunburns, sunbathing vacations, and solarium sessions from members of the NOWAC study were linked to the Janus Cohort members, based on combinations of age, county and time period^33,48^. Continuous variables for lifetime number of sunburns, sunbathing vacations, and solarium sessions were constructed by cumulating annual mean numbers from birth to diagnosis for each case–control set. The rationale for conducting this group-level data linkage between the NOWAC (women only) and the Janus Cohort (men and women) was based on a survey conducted by the Norwegian Cancer Society^49^, showing only small gender differences for sunburns and sunbathing vacations. However, as solarium use was found to be more frequently used by women, group-level data on solarium use was only applied to women^49^.

The linkage to Statistics Norway provided information on occupation and highest attained educational level (none, compulsory, upper secondary, college/university, unknown) at baseline. Occupational UVR exposure at baseline (indoor, mixed, outdoor, unknown) was constructed by categorizing occupational codes according to Alfonso et al.^50^ who used these categories as proxies for occupational sun exposure.

The questions about physical activity and smoking were worded differently in each survey and have been harmonized: physical activity: inactive, low, medium, high, unknown; and smoking status: never, former, current, unknown^40^.

### Data analysis

Continuous variables are presented as means (with standard deviation or range) or medians (with interquartile range) and categorical variables as frequencies (%). Stratified Cox regression was used to estimate hazard ratios (HRs) with 95% confidence intervals (CIs) for the association between 25(OH)D and melanoma risk^43^. Restricted cubic splines with 5 knots (3 knots in stratified analyses) were incorporated into the Cox models to assess the shape of the 25(OH)D-melanoma association using the R package rms^51^. A likelihood ratio test was used to compare the fit of the linear and spline models. We adjusted for BMI, BSA, lifetime ambient UVB, lifetime sunburns, lifetime sunbathing vacations, occupational UVR, education, physical activity, and smoking status in all models. Additional adjustment for solarium use (women only) did not change the results.

Likelihood ratio tests were used to test the statistical significance of interaction between continuous 25(OH)D and ambient UVB of residence (dichotomized into low: north, mid, southwest and high: southeast inland, southeast coast) and BMI (dichotomized into normal weight: BMI < 25 kg/m^2^ and overweight: BMI ≥ 25 kg/m^2^). For ambient UVB of residence, HRs and 95% CI were estimated from stratified analyses, whereas for BMI HRs and 95% CI were computed as linear combinations of the estimated regression coefficients to not loose case–control pairs in the opposite BMI strata. Levels of 25(OH)D in persons with BMI < 25 and ≥ 25 kg/m^2^ were compared by a two sample t-test.

We performed analyses stratified by anatomical site and histological subtype and tested whether 25(OH)D-melanoma associations differed between anatomical sites and between histological subtypes by a contrast test (test for heterogeneity)^52^.

To assess the influence of extreme values we performed sensitivity analyses excluding persons with 25(OH)D below the 2.5 percentile or above the 97.5 percentile (Supplemental Material). To assess the influence of missing values we used multiple imputation with chained equations, assuming missing at random^53^. The imputation model included the outcome and all exposures and adjustment variables. We imputed 30 datasets, and the estimates and standard errors were combined using Rubin's rules (Supplemental Material)^54^.

Tests for significance were two-sided, and the statistical significance level set to < 0.05. Analyses were performed using Stata version 16.1 (StataCorp, College Station, TX, USA) and R version 3.6.1 (https://cran.r-project.org). The R package mice, version 3.6.0, was used for multiple imputation^55^.

### Ethical approval

The study has approval from the Regional Committee for Medical and Health Research Ethics (no. 2014/185).

## Results

Characteristics of the 708 cases and 708 controls are presented in Table 1. Mean 25(OH)D level at baseline was significantly higher among melanoma cases than controls (73.9 vs 70.8 nmol/L, respectively; *P* = 0.03). BMI, BSA, lifetime ambient UVB, sunburns, sunbathing vacations, solarium use, occupational UVR and physical activity were comparable between cases and controls. More cases (22%) held a college/university degree than controls (19%), while a larger proportion of controls (42%) than cases (32%) were current smokers (Table 1). In total, 607 case–control sets had complete information on all covariates (Fig. 1) and their characteristics (Table S1) were largely similar to the characteristics of the whole sample (Table 1), but differed by having comparable proportions of current smokers (cases 32% and controls 33%).

**Table 1:** Characteristics of melanoma cases and controls in the population-based Janus Cohort, Norway, 1972–2009.

Characteristic	Cases (n = 708)	Controls (n = 708)
Males, n (%)	402 (57)	402 (57)
Year of birth, mean (range)	1942 (1922–1965)	1942 (1922–1966)
Age at blood draw (years), mean (range)	42 (22–67)	42 (22–67)
**Season of blood draw, n (%)**		
December–February	151 (21)	151 (21)
March–May	199 (28)	199 (28)
June–August	141 (20)	141 (20)
September–November	217 (31)	217 (31)
25-hydroxyvitamin D (nmol/L), mean (range)^a^	73.9 (19.0–211.4)	70.8 (12.0–196.9)
25-hydroxyvitamin D > 100 nmol/L, n (%)^a^	123 (17)	86 (12)
BMI (kg/m^2^), mean (range)^a^	24.5 (16.1–36.1)	24.6 (16.2–41.8)
BSA (m^2^), mean (range)^a^	1.87 (1.36–2.42)	1.86 (1.36–2.51)
**Ambient UVB of residence at baseline, n (%)**^**b**^		
North	42 (6)	44 (6)
Mid	74 (10)	73 (10)
Southwest	98 (14)	98 (14)
Southeast inland	340 (48)	336 (48)
Southeast coast	154 (22)	157 (22)
Lifetime ambient UVB (kJ × 10^7^), mean (SD)^a,b^	15.7 (2.8)	15.7 (2.9)
Lifetime no. of sunburns, mean (SD)^a,c^	45.6 (5.5)	45.5 (5.5)
Lifetime no. of sunbathing vacations, mean (SD)^a,c^	75.9 (23.4)	75.8 (23.3)
Lifetime no. of solarium sessions, mean (SD)^a,c^	100.8 (40.3)	100.5 (40.2)
**Occupational UVR exposure, n (%)**		
Indoor	407 (57)	434 (61)
Mixed	224 (32)	208 (30)
Outdoor	48 (7)	37 (5)
Unknown	29 (4)	29 (4)
**Education, n (%)**		
None/compulsory	159 (22)	200 (28)
Upper secondary	391 (55)	369 (52)
College/university	157 (22)	136 (19)
Unknown	1 (< 1)	3 (< 1)
**Physical activity, n (%)**		
Inactive	121 (17)	135 (19)
Low	408 (58)	408 (58)
Medium	159 (22)	143 (20)
High	14 (2)	19 (3)
Unknown	6 (< 1)	3 (< 1)
**Smoking status, n (%)**		
Never	280 (40)	231 (33)
Former	183 (26)	164 (23)
Current	227 (32)	296 (42)
Unknown	18 (2)	17 (2)
Age at diagnosis, mean (range)	55.6 (29–75)	–
Time between blood draw and diagnosis, mean (range)	13.9 (2–34)	–
Breslow thickness (mm), median (25th–75th %-tile)^a^	1.0 (0.6–1.95)	–
**T-category (AJCC 8th edition), n (%)**		–
T1 (≤ 1.0 mm)	330 (47)	–
T2 (1.0–2.0 mm)	135 (19)	–
T3 (> 2.0–4.0 mm)	78 (11)	–
T4 (> 4.0 mm)	44 (6)	–
Unknown	121 (17)	–
**Anatomical site, n (%)**		–
Head/neck	73 (10)	–
Trunk	346 (49)	–
Upper limbs	73 (10)	–
Lower limbs	181 (26)	–
Other and not otherwise specified	35 (5)	–
**Histological subtype, n (%)**		–
Superficial spreading melanoma	435 (61)	–
Nodular melanoma	104 (15)	–
Other^d^	27 (4)	–
Not otherwise specified	142 (20)	–

*BMI*  body mass index, *BSA* body surface area, *SD* standard deviation, *UVB* ultraviolet radiation B, *UVR* ultraviolet radiation.

^a^Missing:25-hydroxyvitamin D (n = 17); BMI and BSA (n = 7); lifetime ambient UVB, sunburns, sunbathing vacations, solarium (n = 1).

^b^Based on the UV measurement station closest to county of residence.

^c^Group-level data (age-, county- and time period-specific).

^d^Lentigo malgina melanoma included in other (n = 16).

Table 2 shows associations between 25(OH)D and risk of melanoma for both complete-case analyses and imputed analyses. Little difference was seen between the effect estimates of the complete-case analyses and those of the imputed analyses. The complete-case and the imputed analyses showed HRs of 1.01 (95% CI: 0.99, 1.04) and 1.02 (95% CI: 1.00, 1.04) per 5 nmol/L increase, respectively. No significant effect estimates were found for quintiles or clinical cut points.

**Table 2:** Hazard ratios (HRs) and 95% confidence intervals (CIs) of melanoma according to prediagnostic serum 25-hydroxyvitamin D in the Janus Serum Bank Cohort, Norway, 1972–2009.

	Complete Case^a^			Multiple Imputation^b^	
	Ca/Co	HR^c^	95% CI	HR^c^	95% CI
Continuous (per 5 nmol/L)		1.01	0.99, 1.04	1.02	1.00, 1.04
**Quintiles**					
Quintile 1: < 49.2 nmol/L	109/115	1.00	Referent	1.00	Referent
Quintile 2: 49.2–61.8 nmol/L	122/118	1.05	0.71, 1.55	0.97	0.68, 1.39
Quintile 3: 61.9–74.7 nmol/L	115/124	0.97	0.65, 1.43	1.08	0.75, 1.54
Quintile 4: 74.8–89.2 nmol/L	104/125	0.79	0.53, 1.18	0.88	0.61, 1.27
Quintile 5: > 89.2 nmol/L	157/125	1.34	0.88, 2.03	1.41	0.95, 2.08
**Clinical cut points**					
11–29 nmol/L	14/15	1.09	0.50, 2.38	1.22	0.58, 2.57
30–49 nmol/L	99/102	1.00	Referent	1.00	Referent
50–74 nmol/L	234/241	0.96	0.68, 1.36	1.05	0.76, 1.44
75–212 nmol/L	260/249	1.00	0.70, 1.45	1.12	0.80, 1.58

*BMI* body mass index, *BSA* body surface area, *Ca* cases, *Co* controls, *UVB* = ultraviolet radiation B, *UVR* ultraviolet radiation.

^a^Cases: n = 607; Controls: n = 607.

^b^Cases: n = 708; Controls: n = 708.

^c^Adjusted for BMI, BSA, lifetime ambient UVB, lifetime sunburns, lifetime sunbathing vacations, occupational UVR exposure, education, physical activity, smoking status.

**Figure 2:**
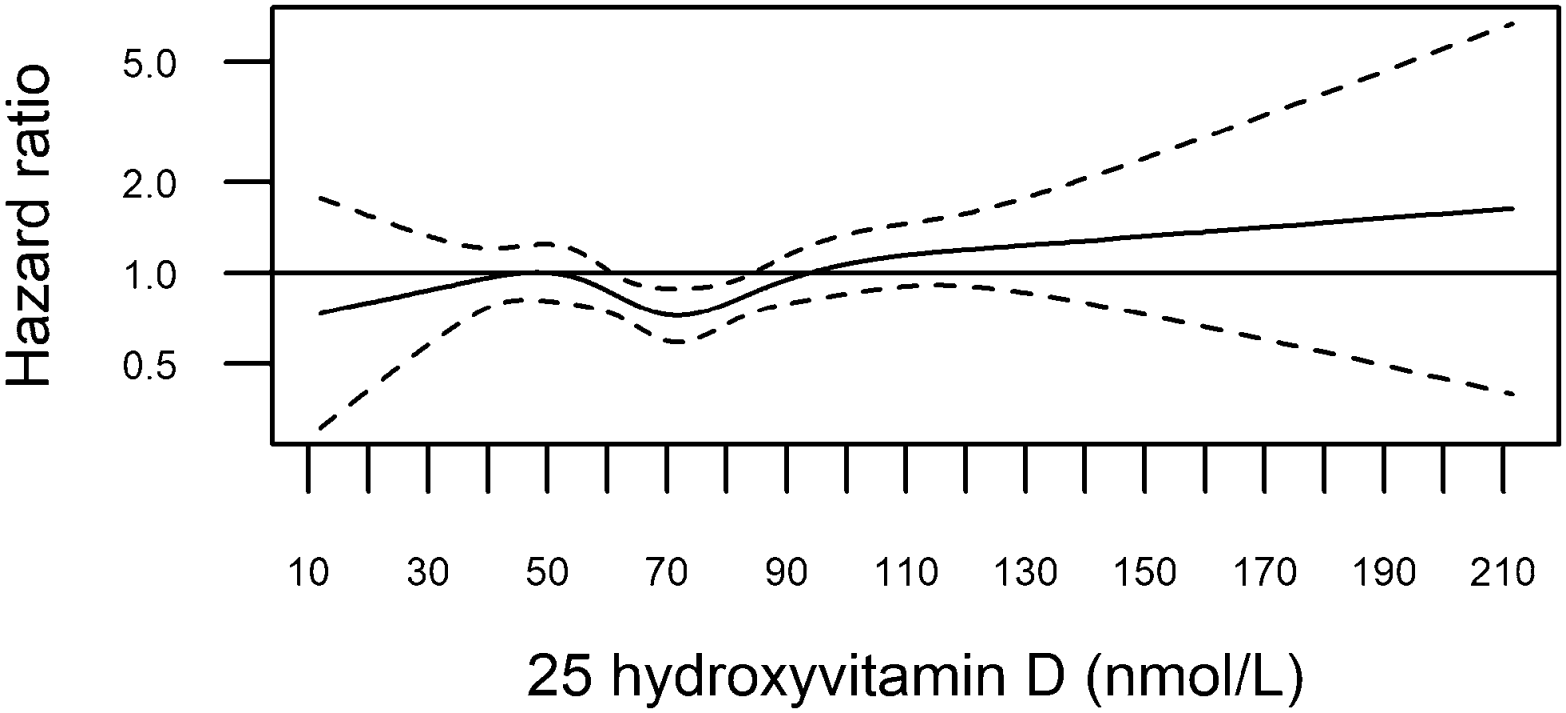
Restricted cubic splines displaying hazard ratios of melanoma with 95% confidence intervals according to prediagnostic serum 25-hydroxyvitamin D in the Janus Serum Bank Cohort, Norway, 1972–2009. Complete case sample: 607 cases and 607 controls. Reference set to 50 nmol/L. Knots located at 36.35, 55.82, 69.83, 85.9, 121.82 nmol/L (first and last at 5 and 95 percentile, the remaining equally spaced), *P* value for non-linearity 0.15. Adjusted for body mass index, body surface area, lifetime ambient ultraviolet (UV)-B, lifetime sunburns, lifetime sunbathing vacations, occupational UV radiation exposure, education, physical activity, smoking status.

In Fig. 2, we modelled the 25(OH)D-melanoma association with restricted cubic splines (complete case). The exposure-risk curve dipped to HRs below 1.00 for 25(OH)D levels between 60 and 85 nmol/L (CI upper bound < 1.00), but the spline model did not show a better fit than the linear model (likelihood ratio test *P* = 0.15). Sensitivity analyses excluding the lowest and highest 25(OH)D values, did not change the shape of the curve materially (Figure S1).

**Figure 3:**
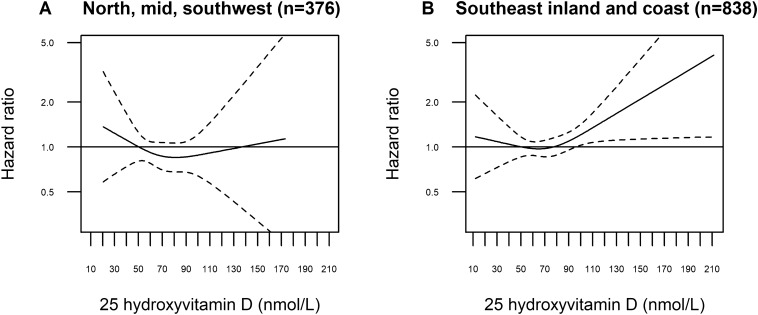
Restricted cubic splines displaying hazard ratios (HRs) of melanoma with 95% confidence intervals according to prediagnostic serum 25-hydroxyvitamin D by ambient UVB of residence in the Janus Serum Bank Cohort, Norway, 1972–2009. **(A)** Splined HRs for north, mid and southwest with knots located at 41.14, 67.48, 106 nmol/L, *P* value for non-linearity 0.456. (**B**) Splined HRs for southeast inland and coast with knots located at 41.43, 70.78, 109.96 nmol/L, *P* value for non-linearity 0.16. **(A,B)** Complete case sample: 607 cases and 607 controls. Reference set to 50 nmol/L. Adjusted for body mass index, body surface area, lifetime ambient ultraviolet (UV)-B, lifetime sunburns, lifetime sunbathing vacations, occupational UV radiation exposure, education, physical activity, smoking status.

Figure 3 shows splines for 25(OH)D and melanoma risk stratified by ambient UVB of residence (complete case). The exposure-risk curve was slightly U-shaped for those residing in the low UVB exposure regions (panel A: north, mid, southwest) but with large uncertainty, whereas a more J-shaped curve was seen among residents with the highest UVB exposure (panel B: southeast). Linear modelling (Table S2, complete case) showed no significant associations and no significant interaction was found (*P*_interaction_ 0.237). Multiple imputation analyses yielded similar results (Table S2). The non-linear models did not show a better fit than the linear models (*P* values 0.46 and 0.16 for lowest and highest ambient UVB of residence, respectively). Sensitivity analyses excluding the lowest and highest 25(OH)D values, made the curve in panel A point slightly more downwards, while the curve in panel B did not change materially (Figure S2).

**Figure 4:**
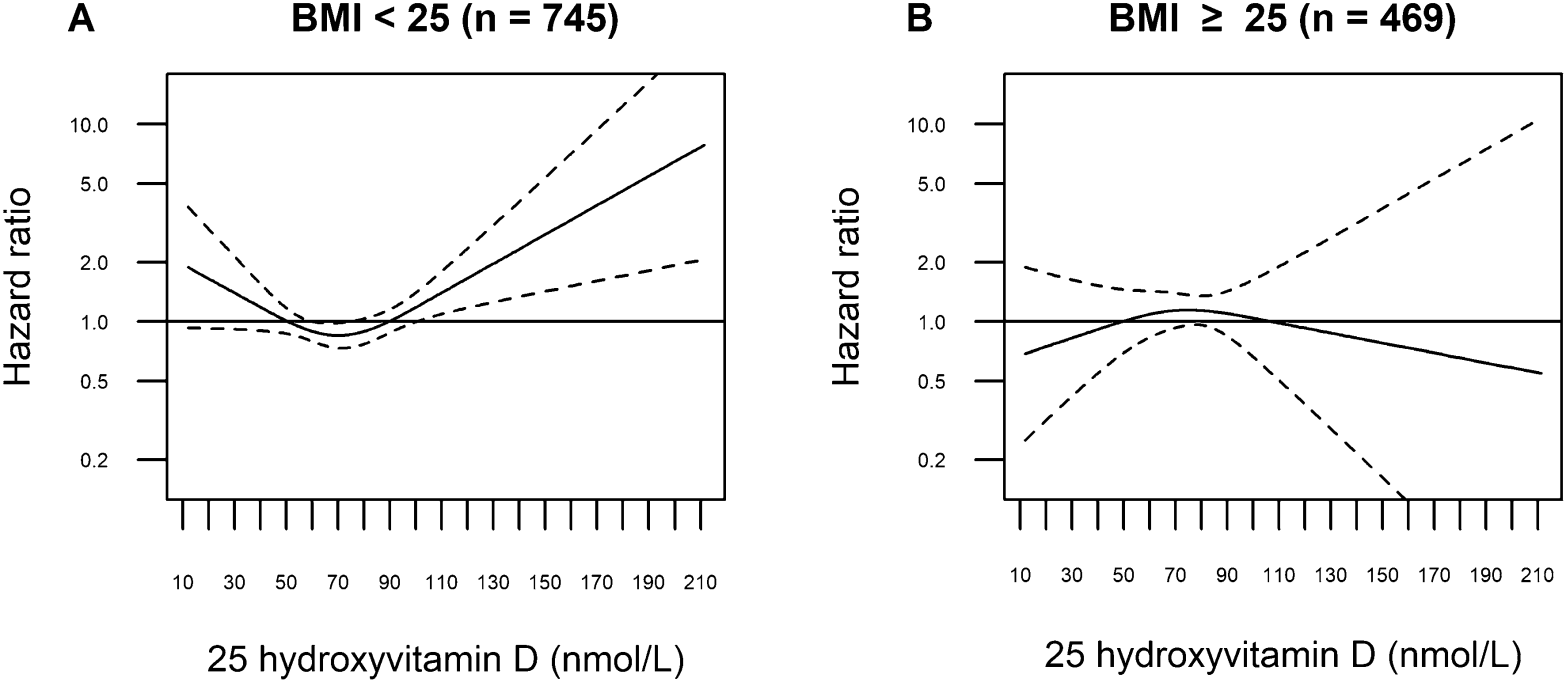
Restricted cubic splines displaying hazard ratios (HRs) of melanoma with 95% confidence intervals according to prediagnostic serum 25-hydroxyvitamin D by body mass index (BMI) in the Janus Serum Bank Cohort, Norway, 1972–2009. The curves are estimated based on linear combinations of the main effect term and the interaction term. Complete case sample: 607 cases and 607 controls. Reference set to 50 nmol/L. Knots located at 41.22, 69.83, 108.43 nmol/L (10, 50 and 90 percentile, the remaining equally spaced). *P*_interaction_ 0.04 and 0.01 for the first (knot 1–2) and second (knot 2–3) curve-segments, respectively. *P* value for non-linearity 0.011. Adjusted for BMI (continuous), body surface area, lifetime ambient ultraviolet (UV)-B, lifetime sunburns, lifetime sunbathing vacations, occupational UV radiation exposure, education, physical activity, smoking status.

Figure 4 shows splines for 25(OH)D and melanoma risk among the normal weight (panel A: BMI < 25 kg/m^2^) and the overweight (panel B: BMI ≥ 25 kg/m^2^). Among the normal weight, the curve indicated a J-shape with reduced risk for 25(OH)D between 60–80 nmol/L (CI upper bound ≈1.00), while among overweight a non-significant curve with a flatter shape was seen. Significant interaction was found in the spline model (P_interaction_ = 0.04 and 0.01 for linear and non-linear associations, respectively). Linear models (Table S3) showed no significant associations and no significant interaction (*P*_interaction_ = 0.73), and the spline model showed a better fit than the linear model (*P* = 0.01). Sensitivity analyses excluding the lowest and highest 25(OH)D values, did not change the shape of the curve in panel A materially, while the curve in panel B pointed slightly more upwards (Figure S3). Mean 25(OH)D was significantly lower in overweight than in normal weight (69.6 *vs* 75.2 nmol/L, respectively; *P* < 0.001).

We also examined the 25(OH)D-melanoma association by anatomic site (Tables S4a and S4b) and found no clear pattern and no significant difference between the sites (*P*_heterogeneity_ = 0.24). When examined by histological subtype (Table S5), no clear pattern was seen and no significant difference was found between the subtypes (*P*_heterogeneity_ = 0.37).

## Discussion

In this prospective analysis, we estimated an overall non-significant positive association between prediagnostic serum 25(OH)D and melanoma risk using linear models. However, when we explored the shape of the association by employing non-linear models, we found an exposure-risk curve suggesting reduced risk of melanoma for 25(OH)D levels between 60 and 85 nmol/L. Indications of exposure-risk curves with a J-shape were seen in sub-analyses of persons with high ambient UVB of residence and BMI < 25 kg/m^2^, although not statistically significant.

Studying the 25(OH)D–melanoma association is challenging due to the possible reverse causation from the melanoma diagnosis itself^31–33^, and due to potentially strong confounding from UVR exposure^35,38^, which is an important source of 25(OH)D and the primary risk factor of melanoma. Our finding of a non-significant positive association is largely in line with other studies examining the 25(OH)D-melanoma association using prediagnostic samples and linear models^34,36–38^. This positive association indicates confounding by UVR as high levels of 25(OH)D are unlikely to cause melanoma^56^.

Our finding of reduced risk for levels between 60 and 85 nmol/L, might be explained by that persons within this range pursue a more healthy lifestyle with moderate UVR exposure and a diet rich on vitamin D^57^. For people living in northern and western Norway, with lower ambient UVB of residence and higher dietary intake of vitamin D^57^, the exposure-risk curve did not rise at higher 25(OH)D levels. The increase in risk seen from 100 nmol/L onwards among persons living in southeastern Norway with the highest ambient UVB exposure of residence, might, however, reflect increased sun exposure. These findings are in line with the results from a comparable Danish cohort that concluded that increasing 25(OH)D levels were associated with increased melanoma risk, and that 25(OH)D is a surrogate marker of UVB exposure^34^. However, high, non-UVB induced, levels of 25(OH)D above 100 nmol/L may also indicate vitamin D treatment due to deficiency among patients^58^.

We found indications of effect modification by BMI in the spline model; the exposure-risk curve among normal weight persons suggested a similar dip (Fig. 4A; 60–80 nmol/L) as in the overall analysis (Fig. 2; 60–85 nmol/L), lending support to the explanation that this might reflect a healthy lifestyle. A flatter shape of the exposure-risk curve was seen among overweight persons, although with large uncertainty for 25(OH)D levels of 100 nmol/L onwards. This might indicate that overweight persons sunbathe less than normal weight persons^59^, and hence that the 25(OH)D-melanoma association is less prone to confounding by UVR in overweight. Our finding that mean 25(OH)D was significantly lower in overweight compared with normal weight, accords with that from another Norwegian cohort^18^, and might be a marker of less UVB exposure^34^ or be due to decreased bioavailability of 25(OH)D among overweight^19^. Importantly, being overweight is per se a possible melanoma risk factor but in comparison with UVR exposure, only a small fraction of the melanoma cases may be attributed to overweight or body size^60^.

In line with previous studies of 25(OH)D and melanoma risk^34–38^, a recent Mendelian randomization study did not find evidence for causal link between genetic determinants of 25(OH)D levels and melanoma risk^61^. Also, we recognize that our spline regressions yielded wide CIs and only showed a significantly better fit to the data for the model including the interaction term with BMI. Further, a J-shaped curve may also reflect underlying co-morbidity resulting in higher risk for lower concentrations of 25(OH)D, or it may be a mathematical side effect of combining a spline model and a Cox model^62^. Although radiation over the whole UV-spectrum (100–400 nm) is considered to cause melanoma in humans^63^, the mechanistic role of UVA and UVB in the melanomagenic process differs. Melanoma induction by UVA is dependent on melanin pigment, while UVB initiates the melanomagenesis independent of melanin pigment^64,65^.

We are therefore precautious about overstating a possible preventive role of 25(OH)D on melanoma risk, and interpret this finding as possible. If, however, 25(OH)D is inversely associated with melanoma risk, it is likely through activation of the 1,25(OH)_2_D receptor^12^, which has been shown to have various anti-proliferative and anti-inflammatory effects^11^. Further, 1,25(OH)_2_ may inhibit growth of neoplasms through cell-cycle arrest^66^, and reduce inflammation and serum cytokine levels through reduction of stress-activated kinase signaling^67,68^.

Our study is the largest prospective study to evaluate the association between 25(OH)D and melanoma risk that includes only invasive and histologically verified incident melanomas from a complete and nationwide cancer registry. To our knowledge, it is also the first study to explore the shape of the 25(OH)D-melanoma association and to conduct sub-analyses by ambient UVB of residence and BMI in a high-latitude population. An important strength is that we only sampled cases and controls without a cancer history, which together with the use of prediagnostic serum samples limit the possibility of reverse causation. Also, our study was nested in the population-based Janus Cohort and linked to the National Population Register securing complete control of loss to follow-up. A major limitation was that our sub-analyses were not adequately powered to exclude or confirm possible medium range associations. Further, we did not have individual information on pigmentary traits and only group level data on sunburns, sunbathing and solarium, which hampered adjustment of these factors and likely resulted in confounding by UVR exposure. Difference in skin color could potentially bias our results, but since the fraction of non-whites in Norway was less than 1% during 1970–1991 (when 97.5% of the Janus Cohort members were recruited), we consider the risk of such bias as small^33^. Serum 25(OH)D levels have been shown to be stable after storage at − 25 °C^69^. A possible time-dependent degradation was compensated for by matching cases and controls on date of blood draw. Difference in time since blood draw might have affected our results, although a recent study from the Janus Cohort, using repeated 25(OH)D measurements in relation to cancer survival, showed that 25(OH)D was stable over time^70^. Quality control samples (results not shown) showed that measurement precision for 25(OH)D was variable, but since matched case–control pairs were placed next to each other on the same batch, intra-batch variability was reduced.

In this large prospective case–control study nested in a population-based cohort, we did not find overall persuasive evidence for an association between 25(OH)D and melanoma risk, although spline curves suggested a possible reduced risk for 25(OH)D serum levels within the normal range (60–85 nmol/L) overall, and in sub-analyses of individuals with BMI < 25 kg/m^2^. The fact that UVR exposure is a common causal factor for both 25(OH)D production and melanoma, may lead to confounding, as was suggested in our results by risk estimates increasing at higher 25(OH)D values in the most sunny parts of the country.

## Supplementary information

Supplementary Information.

## Data Availability

Requests for data sharing/case pooling may be directed to the corresponding author. This project uses third-party data derived from State government registries, which are ultimately governed by their ethics committees and data custodians. Thus, any requests to share these data will be subject to formal approval from each data source used in this project.
