# Supplementary material for: Prediagnostic serum 25-hydroxyvitamin D and melanoma risk
Source: Sci Rep. 2020 Nov 18;10:20129. doi: 10.1038/s41598-020-77155-2 (PMC7676247; doi:10.1038/s41598-020-77155-2)
Supplement: Supplementary file 1 — Supplementary Information. [file 41598_2020_77155_MOESM1_ESM.pdf]

SUPPLEMENTARY MATERIAL

Title: Prediagnostic serum 25-hydroxyvitamin D and melanoma risk

Authors: Jo S Stenehjem[1,2,3], Nathalie C Støer[2], Reza Ghiasvand[1,2], Tom K Grimsrud[2], Ronnie Babigumira[2], Judy R Rees[4,5], Lill Tove Nilsen[6], Bjørn Johnsen[6], Per M Thorsby[7], Marit B Veierød[1], Trude E Robsahm[2]

[1]Oslo Centre for Biostatistics and Epidemiology, Department of Biostatistics, University of Oslo, Oslo, Norway
[2]Department of Research, Cancer Registry of Norway, Oslo, Norway
[3]Division of Emergencies and Critical Care, Oslo University Hospital, Norway
[4]New Hampshire State Cancer Registry, Lebanon, NH, USA
[5]Department of Epidemiology, Geisel School of Medicine at Dartmouth, Lebanon, NH, USA
[6]Norwegian Radiation Protection Authority, Østerås, Norway.
[7]Hormone Laboratory, Department of Medical Biochemistry, Oslo University Hospital, Oslo Norway

Content:

- Supplementary tables and information
    - Table S1
    - Multiple imputations
    - Table S2
    - Table S3
    - Table S4a
    - Table S4b
    - Table S5
    - Figure S1
    - Figure S2
    - Figure S3

**Table S1.** Characteristics of Melanoma Cases and Controls in the Population-based Janus Cohort, Norway, 1972–2009, Complete Case Sample.		
**Characteristic**	**Cases (n=607)**	**Controls (n=607)**
Males, n (%)	370 (61)	370 (61)
Year of birth, mean (range)	1942 (1922–1960)	1942 (1922–1959)
Age at blood draw (years), mean (range)	42 (22–67)	42 (22–67)
Season of blood draw, n (%)		
December-February	129 (21)	129 (21)
March-May	168 (28)	168 (28)
June-August	123 (20)	123 (20)
September-November	187 (31)	187 (31)
25-hydroxyvitamin D (nmol/L), mean (range)	74.1 (19.0–211.4)	72.0 (12.0–196.9)
BMI (kg/m^2), mean (range)	24.5 (16.1–36.1)	24.6 (16.2–41.8)
BSA (m^2), mean (range)	1.89 (1.43–2.42)	1.87 (1.39–2.51)
Ambient UVB of residence region at baseline, n (%)[a]		
North	34 (6)	36 (6)
Mid	72 (12)	71 (12)
Southwest	82 (13)	82 (13)
Southeast inland	280 (46)	279 (46)
Southeast coast	139 (23)	139 (23)
Lifetime ambient UVB of residence, mean (SD)[a]	15.8 (2.9)	15.8 (2.9)
Lifetime no. of sunburns, mean (SD)[b]	45.7 (26–59)	45.7 (26–59)
Lifetime no. of sunbathing vacations, mean (SD)[b]	75.8 (31–149)	75.8 (34–149)
Lifetime no. of solarium sessions, mean (SD)[b]	105.1 (22–211)	104.7 (16–218)
Occupational UVR exposure, n (%)		
Indoor	358 (59)	387 (64)
Mixed	205 (34)	185 (30)
Outdoor	44 (7)	35 (6)
Education, n (%)		
None/compulsory	128 (21)	162 (27)
Upper secondary	340 (56)	327 (54)
College/university	139 (23)	118 (19)
Physical activity, n (%)		
Inactive	100 (17)	112 (18)
Low	354 (58)	347 (57)
Medium	140 (23)	131 (22)
High	13 (2)	17 (3)
Smoking status, n (%)		
Never	245 (40)	257 (42)
Former	166 (28)	149 (25)
Current	196 (32)	201 (33)
Age at diagnosis, mean (range)	56 (35–75)	—
Time between blood draw and diagnosis, mean (range)	14.1 (2–34)	—
Breslow thickness (mm), median (25th-75th %-tile)	1 (0.6–1.95)	—
T-category (AJCC 8th edition), n (%)		—
T1 (≤1.0 mm)	285 (47)	—
T2 (1.0–2.0 mm)	117 (19)	—
T3 (>2.0–4.0 mm)	64 (11)	—
T4 (>4.0 mm)	41 (7)	—
Unknown	100 (16)	—
Anatomical site, n (%)		—
Head/neck	58 (10)	—
Trunk	307 (51)	—
Upper limbs	62 (10)	—
Lower limbs	148 (24)	—
Other and not otherwise specified	32 (5)	—
Histological subtype, n (%)		—
Superficial spreading melanoma	370 (61)	—
Nodular melanoma	90 (15)	—
Other[c]	24 (4)	—
Not otherwise specified	123 (20)	—
Abbreviations: BMI = body mass index; BSA = body surface area; SD = standard deviation; UVB = ultraviolet radiation-B; UVR = ultraviolet radiation.		
[a]Based on the UV measurement station closest to county of residence.		
[b]Group-level data (age-, county- and time period-specific).		
[c]Lentigo malgina melanoma included in other (n=16)		

Multiple imputations

Complete case analysis (n=1214)
As shown in Figure 1, the study sample for our complete case analysis included 607 melanoma case-control pairs (complete case-control pairs were excluded when either only the case or the control had missing on a variable), for which the results in Tables 2, S1, S2, S3, S4a and S5, and Figures 2-4 and S1-S2 are shown.

Multiple imputation analysis (n=1416)
As shown in Figure 1, the study sample for our multiple imputation analyses included 708 melanoma case-control pairs. In this sample, 9% (n=134) had missing data on one or more of the following variables:

- 25-hydroxyvitamin D: n=17
- BMI/BSA: n=7
- Lifetime UVB: n=1
- Sunburns: n=1
- Sunbathing vacations: n=1
- Solarium: n=1
- Occupational UVR: n=58
- Education: n=4
- Physical activity: n=9
- Smoking status: n=35

Assuming missing at random, we used multiple imputation with chained equations as described by White et al. (2011). The imputation procedure followed 2-steps;

1. We considered the following variables as likely to predict missingness: sex, ambient UVB of residential region, case status, age at blood draw, season for blood draw and used these variables as predictors together with the variables from the estimation model (*i.e.* 25(OH)D, BMI, BSA, lifetime sunbathing vacations, lifetime sunburns, lifetime UVB at residence, occupational UVR, physical activity, smoking status, education) to simultaneously predict missing data on all variables. Predictive mean matching and multinomial logit models were used to predict missing data for continuous and categorical variables, respectively.[1-2] The imputation model was ran 30 times, using the rule of thumb of running at least as many times as the percentage of missing data.[3] Estimates and standard errors were combined using Rubin's rules.[4] Convergence was checked and the imputed values found reasonable. Similar limits for quintile cut-off points as in the complete case analyses were used.
2. After step 1, we ran analyses on the imputed dataset (n=1416) as shown in Tables 2, S2, S3a, S3b and S4

References
1. Little R. J. A. Missing Data Adjustments in Large Surveys. *Journal of Business Economics and Statistics* **6**, 287-301 (1988).
2. Buuren, S. v. & Groothuis-Oudshoorn, K. mice: Multivariate imputation by chained equations in R. *Journal of statistical software*, 1-68 (2010).
3. White I. R., Royston P. & Wood A. M. Multiple imputation using chained equations: Issues and guidance for practice. *Statistics in medicine,* **30,** 377-399 (2011).
4. Rubin, D. B. *Multiple imputation for nonresponse in surveys*. Vol. 81 (John Wiley & Sons, 2004).

Table S2. Hazard Ratios (HRs) and 95% Confidence Intervals (CIs) of Melanoma according to Prediagnostic Serum 25-hydroxyvitamin D by Ambient UVB of Residence at Baseline in the Janus Serum Bank Cohort, Norway, 1972–2009

	**Complete Case[a]**						**Multiple Imputation[b]**			
	**North, Mid and Southwest**			**Southeast Inland and Coast**			**North, Mid and Southwest**		**Southeast Inland and Coast**	
	**Ca/Co[a]**	**HR[c]**	**95% CI**	**Ca/Co[a]**	**HR[c]**	**95% CI**	**HR[c]**	**95% CI**	**HR[c]**	**95% CI**
Continuous (per 5 nmol/L)[d]		0.99	0.94, 1.04		1.02	0.99, 1.06	0.99	0.94, 1.04	1.03	1.00, 1.06
Tertiles										
Tertile 1: < 58.5 nmol/L	70/65	1.00	Referent	129/128	1.00	Referent	1.00	Referent	1.00	Referent
Tertile 2: 58.5–79.8 nmol/L	60/63	0.75	0.44, 1.28	120/141	0.81	0.55, 1.17	0.84	0.51, 1.39	0.97	0.68, 1.37
Tertile 3: > 79.8 nmol/L	58/60	0.73	0.39, 1.37	170/150	1.16	0.78, 1.71	0.80	0.45, 1.43	1.29	0.90, 1.87
Clinical cut points										
11–29 nmol/L	4/5	0.63	0.13, 3.05	10/10	1.32	0.52, 3.39	0.78	0.17, 3.63	1.27	0.52, 3.12
30–49 nmol/L	34/31	1.00	Referent	65/71	1.00	Referent	1.00	Referent	1.00	Referent
50–74 nmol/L	81/85	0.71	0.35, 1.43	153 156	1.04	0.69, 1.56	0.93	0.48, 1.80	1.07	0.74, 1.55
75–212 nmol/L	69/67	0.73	0.35, 1.56	191/182	1.12	0.73, 1.72	0.94	0.48, 1.86	1.19	0.80, 1.78

Abbreviations: BMI = body mass index; BSA = body surface area; Ca = cases; Co = controls; UVB = ultraviolet radiation B; UVR = ultraviolet radiation.

[a]Cases: n=607; Controls: n=607 (missing data excluded)

[b]Cases: n=708; Controls: n=708 (missing data imputed)

[c]Adjusted for BMI, BSA, lifetime sunbathing vacations, lifetime sunburns, lifetime UVB at residence, occupational UVR, education, physical activity, smoking status.

[d]P-value for interaction between 25(OH)D (continuous) and ambient UVB residence in complete case analysis: 0.237

Table S3. Hazard Ratios (HRs) and 95% Confidence Intervals (CIs) of Melanoma according to Prediagnostic Serum 25-hydroxyvitamin D by Body Mass Index (BMI) in the Janus Serum Bank Cohort, Norway, 1972–2009

	**Complete Case[a]**					
	**BMI <25 kg/m²**			**BMI ≥25 kg/m²**		
	**Ca/Co[a]**	**HR[b,c]**	**95% CI**	**Ca/Co[a]**	**HR[b,c]**	**95% CI**
Continuous (per 5 nmol/L)[d]		1.02	0.99, 1.05		1.00	0.96, 1.04
Quintiles						
Quintile 1: < 49.2 nmol/L	61/59	1.00	Referent	48/56	1.00	Referent
Quintile 2: 49.2–61.8 nmol/L	72/66	1.10	0.65, 1.87	50/52	0.99	0.55, 1.76
Quintile 3: 61.9–74.7 nmol/L	64/82	0.73	0.44, 1.23	51/42	1.50	0.82, 2.74
Quintile 4: 74.8–89.2 nmol/L	63/83	0.68	0.40, 1.14	41/42	1.06	0.57, 2.00
Quintile 5: > 89.2 nmol/L	113/82	1.39	0.83, 2.32	44/43	1.28	0.68, 2.43
Clinical cut points						
11–29 nmol/L	10/6	2.17	0.71, 6.63	4/9	0.47	0.14, 1.65
30–49 nmol/L	54/53	1.00	Referent	45/49	1.00	Referent
50–74 nmol/L	133/149	0.86	0.54, 1.37	101/92	1.11	0.66, 1.85
75–212 nmol/L	176/164	1.00	0.63, 1.59	84/85	0.99	0.56, 1.72

Abbreviations: BMI = body mass index; BSA = body surface area; Ca = cases; Co = controls; UVB = ultraviolet radiation B; UVR = ultraviolet radiation.

[a]Cases: n=607; Controls: n=607 (missing data excluded)

[b]Adjusted for BMI, BSA, lifetime sunbathing vacations, lifetime sunburns, lifetime UVB at residence, occupational UVR, education, physical activity, smoking status.

[c]HRs and 95% CIs computed as linear combinations of the estimated regression coefficients

[d]P-value for interaction between 25(OH)D (continuous) and BMI: 0.729

Table S4a. Hazard Ratios (HRs) and 95% Confidence Intervals (CIs) of Melanoma according to Prediagnostic Serum 25-hydroxyvitamin D by anatomic site (Complete Case Sample) in the Janus Serum Bank Cohort, Norway, 1972–2009

	Head and neck (n=58)			Trunk (n=307)			Upper limbs (n=62)			Lower limbs (n=148)		
	**Ca/Co[a]**	**HR[b]**	**95% CI**	**Ca/Co[a]**	**HR[b]**	**95% CI**	**Ca/Co[a]**	**HR[b]**	**95% CI**	**Ca/Co[a]**	**HR[b]**	**95% CI**
Continuous (per 5 nmol/L)[c]		1.07	0.98, 1.17		1.00	0.96, 1.04		0.93	0.82, 1.05		1.03	0.98, 1.09
Tertiles												
Tertile 1: < 58.5 nmol/L	19/16	1.00	Referent	105/103	1.00	Referent	15/18	1.00	Referent	50/48	1.00	Referent
Tertile 2: 58.5–79.8 nmol/L	11/20	0.41	0.09, 1.91	93/102	0.79	0.52, 1.21	25/20	1.00	0.24, 4.19	40/54	0.62	0.31, 1.23
Tertile 3: > 79.8 nmol/L	28/22	1.21	0.35, 4.24	109/102	0.94	0.59, 1.5	22/24	0.35	0.07, 1.84	58/46	1.16	0.59, 2.26
Clinical cut points												
11–29 nmol/L	2/1	1.23	0.03, 44.39	9/9	1.12	0.39, 3.2	1/1	0.11	0.00, 5.21	1/3	0.22	0.02, 2.55
30–49 nmol/L	11/8	1.00	Referent	53/54	1.00	Referent	6/9	1.00	Referent	24/27	1.00	Referent
50–74 nmol/L	16/21	0.55	0.14, 2.27	116/123	0.92	0.57, 1.51	30/24	0.49	0.07, 3.57	58/62	1.14	0.53, 2.44
75–212 nmol/L	29/28	0.67	0.17, 2.62	129/121	0.98	0.58, 1.65	25/28	0.17	0.02, 1.34	65/56	1.51	0.68, 3.34

Abbreviations: BMI = body mass index; BSA = body surface area; Ca = cases; Co = controls; UVB = ultraviolet radiation B; UVR = ultraviolet radiation.

[a]Cases: n=575; Controls: n=575 (missing data excluded and 32 case-control pairs of other/not otherwise specified sites not studied)

[b]Adjusted for BMI, BSA, lifetime sunbathing vacations, lifetime sunburns, lifetime UVB at residence, occupational UVR, education, physical activity, smoking status.

[c]P-value for heterogeneity between anatomic sites: 0.235

Table S4b. Hazard Ratios (HRs) and 95% Confidence Intervals (CIs) of Melanoma according to Prediagnostic Serum 25-hydroxyvitamin D by anatomic site (Multiple Imputations[a]) in the Janus Serum Bank Cohort, Norway, 1972–2009

	**Head and neck (n=73)**		**Trunk (n=346)**		**Upper limbs (n=73)**		**Lower limbs (n=181)**	
	**HR[b]**	**95% CI**	**HR[b]**	**95% CI**	**HR[b]**	**95% CI**	**HR[b]**	**95% CI**
Continuous (per 5 nmol/L)	1.05	0.97, 1.13	1.01	0.97, 1.04	0.94	0.84, 1.05	1.05	1.00, 1.11
Tertiles								
Tertile 1: < 58.5 nmol/L	1.00	Referent	1.00	Referent	1.00	Referent	1.00	Referent
Tertile 2: 58.5–79.8 nmol/L	0.49	0.15, 1.58	0.93	0.63, 1.38	1.26	0.33, 4.80	0.82	0.44, 4.80
Tertile 3: > 79.8 nmol/L	0.98	0.38, 2.53	1.06	0.68, 1.64	0.48	0.10, 2.23	1.42	0.76, 2.23
Clinical cut points								
11–29 nmol/L	1.93	0.11, 33.6	1.30	0.48, 3.57	0.24	0.00, 16	0.26	0.02, 2.93
30–49 nmol/L	1.00	Referent	1.00	Referent	1.00	Referent	1.00	Referent
50–74 nmol/L	0.60	0.19, 1.87	1.00	0.63, 1.56	1.07	0.21, 5.38	1.21	0.61, 2.39
75–212 nmol/L	0.78	0.26, 2.29	1.05	0.64, 1.71	0.32	0.06, 1.82	1.87	0.90, 3.87

Abbreviations: BMI = body mass index; BSA = body surface area; Ca = cases; Co = controls; UVB = ultraviolet radiation B; UVR = ultraviolet radiation.

[a]Cases: n=708; Controls: n=708 (missing data imputed and 35 case-control pairs of other/not otherwise specified sites not studied)

[b]Adjusted for BMI, BSA, lifetime sunbathing vacations, lifetime sunburns, lifetime UVB at residence, occupational UVR, education, physical activity, smoking status.

**Table S5.** Hazard Ratios (HRs) and 95% Confidence Intervals (CIs) of Melanoma according to Prediagnostic Serum 25-hydroxyvitamin D by Histological Subtype in the Janus Serum Bank Cohort, Norway, 1972–2009										
	**Complete Case[a]**						**Multiple Imputation[b]**			
	**SSM (n=370)**			**NM (n=90)**			**SSM (n=435)**		**NM (n=104)**	
	**Ca/Co[a]**	**HR[c]**	**95% CI**	**Ca/Co[a]**	**HR[c]**	**95% CI**	**HR[c]**	**95% CI**	**HR[c]**	**95% CI**
Continuous (per 5 nmol/L)[a]		1.01	0.98, 1.05		1.05	0.98, 1.14	1.02	0.99, 1.05	1.05	0.98, 1.13
Tertiles										
Tertile 1: < 58.5 nmol/L	114/110	1.00	Referent	31/36	1.00	Referent	1.00	Referent	1.00	Referent
Tertile 2: 58.5–79.8 nmol/L	119/129	0.82	0.55, 1.23	21/25	0.91	0.36, 2.30	0.98	0.68, 1.43	1.00	0.43, 2.31
Tertile 3: > 79.8 nmol/L	137/131	0.90	0.58, 1.38	38/29	2.34	0.92, 5.92	1.05	0.70, 1.59	2.02	0.90, 4.55
Clinical cut points										
11–29 nmol/L	6/6	1.39	0.38, 5.04	4/5	0.70	0.14, 3.52	1.73	0.51, 5.85	0.78	0.17, 3.52
30–49 nmol/L	60/64	1.00	Referent	14/16	1.00	Referent	1.00	Referent	1.00	Referent
50–74 nmol/L	145/149	1.03	0.65, 1.62	31/35	1.30	0.40, 4.17	1.18	0.77, 1.80	1.18	0.44, 3.13
75–212 nmol/L	159/151	1.03	0.64, 1.67	41/34	2.17	0.63, 7.52	1.21	0.77, 1.91	1.76	0.64, 4.88
Abbreviations: BMI = body mass index; BSA = body surface area; Ca = cases; Co = controls; NM = nodular melanoma; SSM = superficial spreading melanoma; UVB = ultraviolet radiation B; UVR = ultraviolet radiation.										
[a]Cases: n=460; Controls: n=460 (missing data excluded and 147 case-control pairs of other/not otherwise specified sites not studied); P-value for heterogeneity between histological subtypes in complete case analyses: 0.365										
[b]Cases: n=539; Controls: n=539 (missing data imputed and 169 case-control pairs of other/not otherwise specified sites not studied)										
[c]Adjusted for BMI, BSA, lifetime sunbathing vacations, lifetime sunburns, lifetime UVB at residence, occupational UVR, education, physical activity, smoking status.										

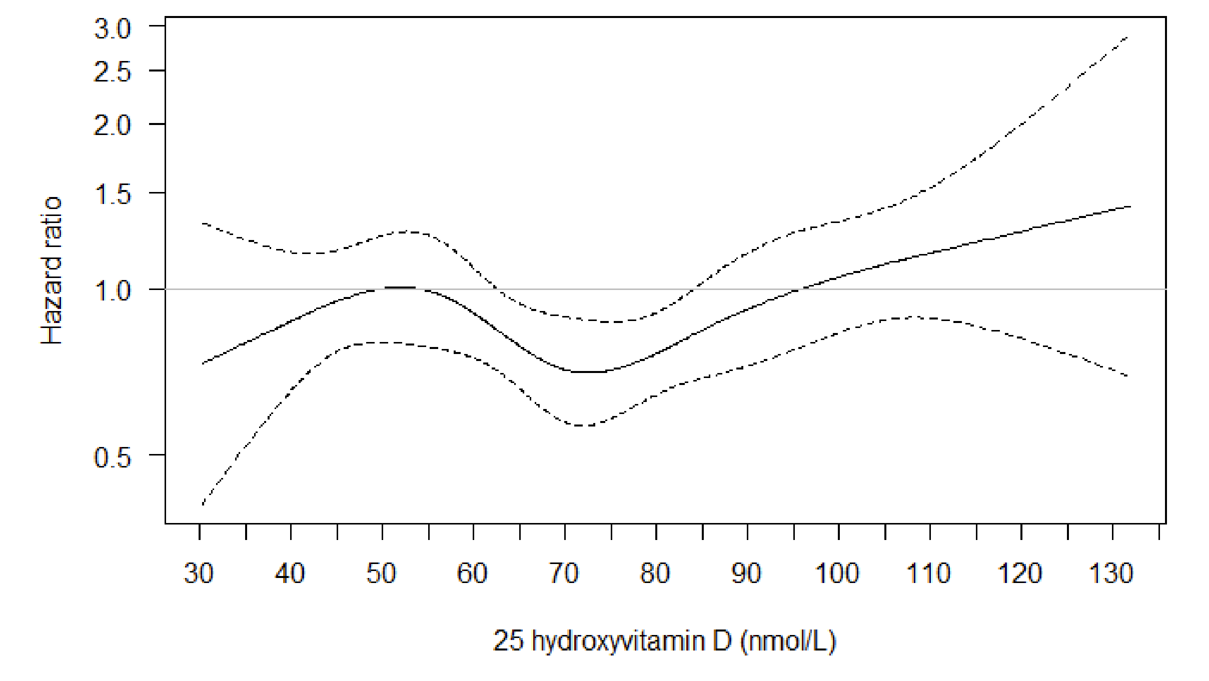

Figure S1. Sensitivity analyses excluding persons with serum 25-hydroxyvitamin D (25(OH)D) below the 2.5 percentile or above the 97.5 percentile. Restricted cubic splines displaying hazard ratios of melanoma with 95% confidence intervals according to prediagnostic 25(OH)D levels in the Janus Serum Bank Cohort. Reference set to 50 nmol/L. Knots located at 36.35, 55.82, 69.83, 85.9, 121.82 nmol/L (first and last at 5 and 95 percentile, the remaining equally spaced), *P* value for non-linearity 0.15. Adjusted for body mass index, body surface area, lifetime ambient ultraviolet (UV)-B, lifetime sunburns, lifetime sunbathing vacations, occupational UV radiation exposure, education, physical activity, smoking status.

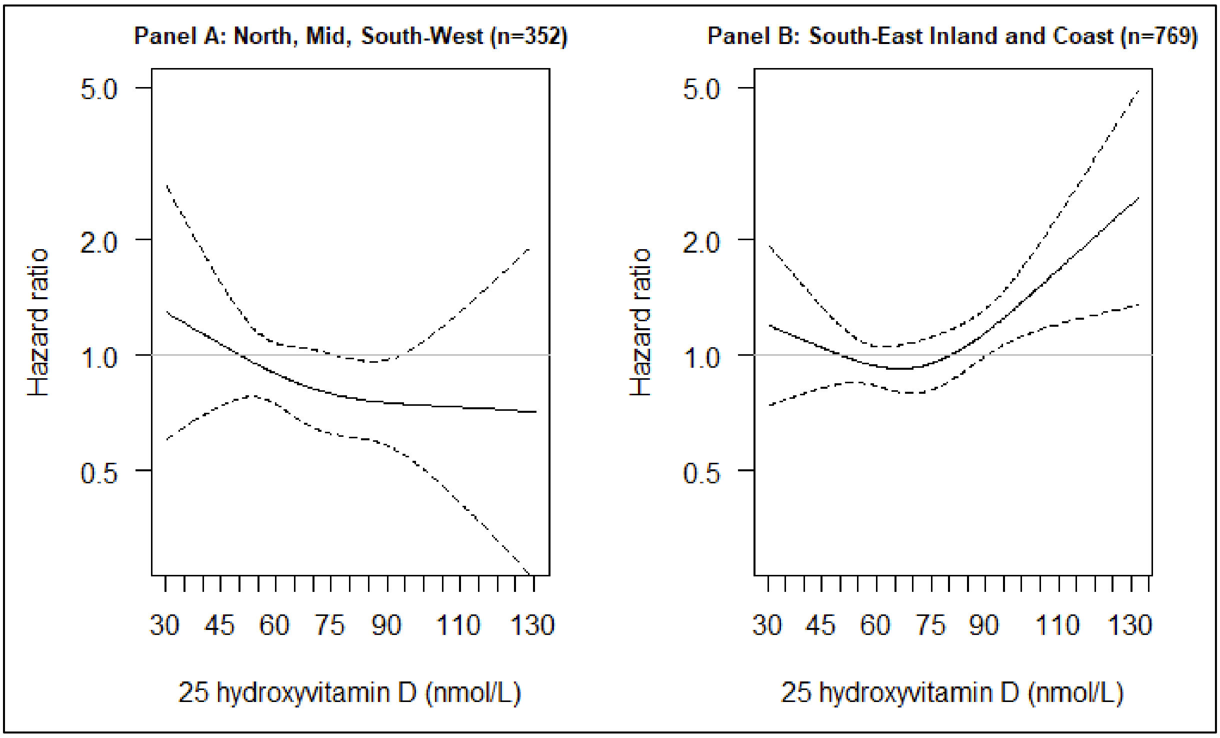

Figure S2. Sensitivity analyses excluding persons with serum 25-hydroxyvitamin D (25(OH)D) below the 2.5 percentile or above the 97.5 percentile. Restricted cubic splines displaying hazard ratios of melanoma with 95% confidence intervals according to prediagnostic 25(OH)D levels in the Janus Serum Bank Cohort by ambient UVB of residence. Panel A: Splined HRs for north, mid and southwest with knots located at 41.14, 67.48, 106 nmol/L, *P* value for non-linearity 0.46. Panel B: Splined HRs for southeast inland and coast with knots located at 41.43, 70.78, 109.96 nmol/L, *P* value for non-linearity 0.16. Panel A and B: Reference set to 50 nmol/L. Adjusted for body mass index, body surface area, lifetime ambient ultraviolet (UV)-B, lifetime sunburns, lifetime sunbathing vacations, occupational UV radiation exposure, education, physical activity,

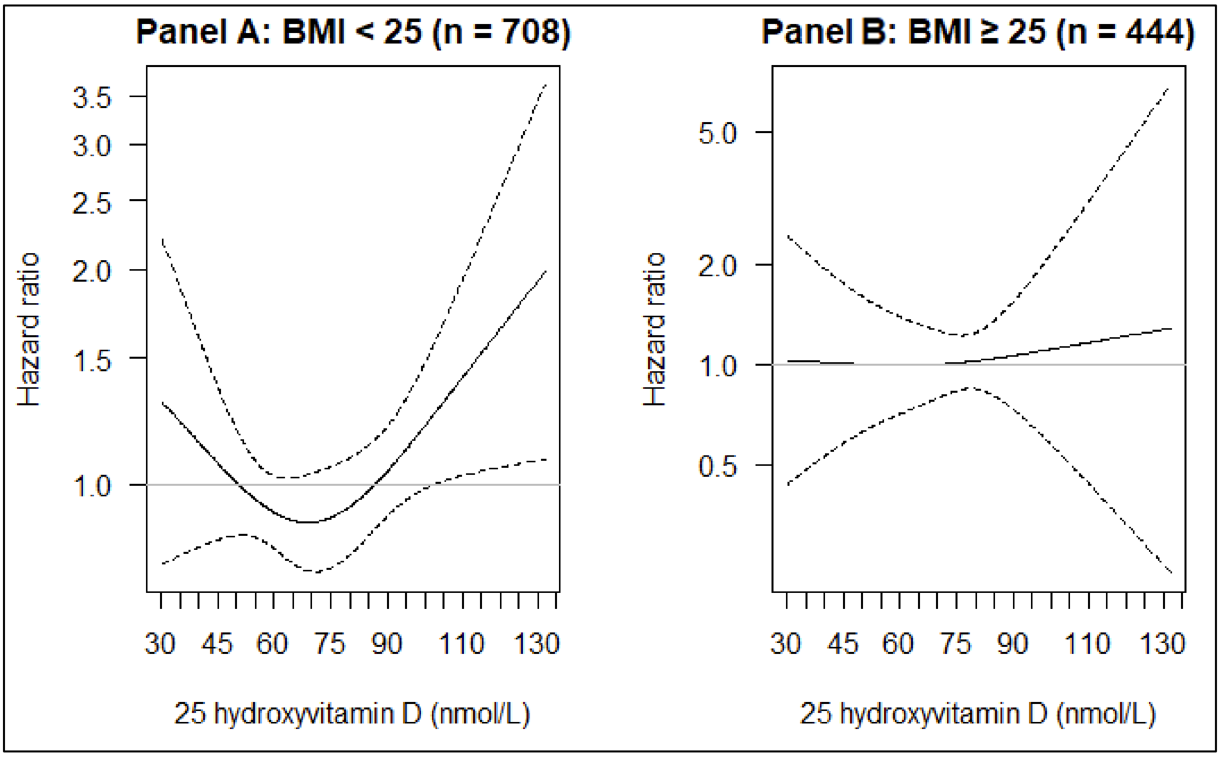

Figure S3. Sensitivity analyses excluding persons with serum 25-hydroxyvitamin D (25(OH)D) below the 2.5 percentile or above the 97.5 percentile. Restricted cubic splines displaying hazard ratios of melanoma with 95% confidence intervals according to prediagnostic 25(OH)D in the Janus Serum Bank Cohort levels by body mass index (BMI). Reference set to 50 nmol/L. Knots located at 41.22, 69.83, 108.43 nmol/L (10, 50 and 90 percentile, the remaining equally spaced), *P* value for non-linearity 0.01. Adjusted for BMI (continuous), body surface area, lifetime ambient ultraviolet (UV)-B, lifetime sunburns, lifetime sunbathing vacations, occupational UV radiation exposure, education, physical activity, smoking status.
